# Impact of a home-based nutritional intervention program on nutritional status of preschool children: a cluster randomized controlled trial

**DOI:** 10.1186/s12889-022-14900-4

**Published:** 2023-01-07

**Authors:** Ansuya B, Baby S. Nayak, Unnikrishnan B, Ravishankar N, Shashidhara Y. N, Suneel C. Mundkur

**Affiliations:** 1 Department of Community Health Nursing, Manipal College of Nursing, Manipal Academy of Higher Education, Manipal, Karnataka India; 2 Department of Child Health Nursing, Manipal College of Nursing, Manipal Academy of Higher Education, Manipal, Karnataka India; 3 Kasturba Medical College, Mangalore, Manipal Academy of Higher Education, Manipal, Karnataka India; 4 Department of Biostatistics, Vallabhbhai Patel Chest Institute, New Delhi, India; 5 Department of Community Health Nursing, Manipal College of Nursing, Manipal Academy of Higher Education, Manipal, Karnataka India; 6 Department of Pediatrics, Kasturba Medical College, Manipal, Manipal Academy of Higher Education, Manipal, Karnataka India

**Keywords:** Efficacy, Home-based diet, Malnutrition, Under-five children, Management, Underweight

## Abstract

**Background:**

Undernutrition in under-five children remains a worldwide health issue and is considered one of the leading causes of increased morbidity and mortality. This study aims to assess the impact of home-based nutritional intervention on the nutritional status of preschool children living in rural areas of South India.

**Methods:**

A single-blinded cluster randomized controlled trial evaluated the impact of the intervention, with weight gain as the primary outcome. A cluster of 12 villages was randomized to intervention or control arms. A total of 253 underweight preschool children from 12 clusters (villages) were randomized to intervention (*n* = 127) and control arm (*n* = 126). The intervention was composed of a health-teaching program and a demonstration of nutritious food preparation in addition to the regular services provided at the Anganwadi centers. The control arm received only standard routine care provided in the Anganwadi centre. The anthropometric assessment was carried out at the baseline and every month for a year.

**Result:**

A significant increase in the mean weight kilograms was noted in the intervention group (11.9 ± 0.98 to 13.78 ± 0.89) compared to the control group (11.8 ± 1.03 to 12.96 ± 0.88). In the intervention group, at the baseline, 41.5% were moderately malnourished (> − 2SD—3SD), which decreased to 24% at the end of the year. Similarly, severe malnutrition decreased from 8.69 to 3.16%, while 20.5% of malnourished children achieved normal nutritional status. In the control group, undernourished children demonstrated minimal changes in nutritional status. Analysis of repeated measures of ANOVA results between the intervention and control groups on weight measurements (F (1, 251) = 15.42, p .001) and height measurements (F (2, 1258) = 1.540, p .001) revealed statistical significance.

**Conclusion:**

The nutritional status of preschool children is found to be improved by home-based intervention, which includes training mothers or caregivers in planning and preparing healthy nutritious diets, providing timely care, and gaining an understanding and knowledge of the nutritional status along with regular home-based diet preparation.

**Trial registration:**

ctri@gov.in CTRI/2017/03/008273 [Registered on: 31/03/2017].

## Background

The nutritional status of children is one of the known indicators for the economic development of a country [1]. 29% of the world’s population is comprised of children under the age of five and an estimated 121.3 million are under five in India. Regarding the under-five mortality rate, approximately 80% of children under five in the world died in just two regions – Sub-Saharan Africa (50%) and South Asia (30%) [2]. According to National Institution for Transforming India, Government of India, the under-five mortality rate in India is 43/1000 live births in 2015 & 39.4/1000 live births in 2017, & in Karnataka, it is 28/1000 live births [3].

Globally, malnutrition is the single cause of nearly 45% of all deaths among under-five children, which accounts for the loss of roughly three million lives of children in a year. Due to malnutrition, the danger of children dying of common infections has intensified. Malnutrition increases the severity and recurring incidence of such common infections. It also slows down the healing process considerably, hence making recovery a difficult process [4].

In India, malnutrition is still soaring. In the third and fourth National Health Surveys, the ministry recounted the trends of malnutrition. The third National Survey (2005–06) found 42.5% of under-fives in India to be underweight, 26.2% wasted and 48% stunted [5]. The National Survey of 2015–16 revealed that 35.7% of children under the age of five were underweight, 28.5% were wasted, and 38.4% were stunted [6]. Several improvement programs were implemented, and subsequently, there has been a decrease in the number of children under the age of five who are underweight or stunted, but it is still apparent that the status of children getting wasted has only increased rather than declined [7].

In India, to achieve the target of reducing malnutrition, many more nutritional programs were implemented and some programs are still in progress, but the burden of under-nutrition in children has not been reduced significantly. For severe acute malnutrition, the WHO recommended home-based management with Ready-to-Use Therapeutic Food (RUTF) [4].

Numerous government projects have been implemented over the years such as the Integrated Child Development Services (ICDS), the National Health Mission, the Janani Suraksha Yojana, the Matritva Sahyog Yojana, the Mid-Day Meal Scheme, and the National Food Security Mission, etc. aimed at improving the nutritional status of the country [8]. Still, over the years, the burden of malnutrition has continued to exist. The Sustainable Development Goals (SDGs) adopted by the United Nations (UN) in 2015 included malnutrition (stunting) reduction as one of their goals (SDG 2.2) [9]. Randomized control trial evaluated the home-based therapeutic food was more effective in terms of better acceptability, better palatability, affordability, increased frequency of feeding, and less difficulty in making [10]. The effect of nutritional intervention with a cooked meal for 175 days had a significant reduction in wasting among preschoolers [11]. Keeping this in mind the present study aimed to assess the impact of a home-based nutrition intervention program on the nutritional status of preschool children.

## Methods

Study design: A community-based cluster randomized controlled trial (cRCT) was conducted in the Udupi district of Karnataka, India.

### Study area and participants

This study was conducted among moderate (weight-for-age < −2SD to -3SD z score), and severely underweight (weight-for-age < −3SD) preschool children between three and five years of age and their mothers registered in Anganwadi centers of Udupi district, Karnataka, India.

### Sample size

The sample size for the study was calculated considering weight gain as the primary outcome variable, 80% power, 5% level of significance, an attrition rate of 10%, weight gain in the control group of 10%, and weight gain in the intervention group as 25%. The required sample size was 234; 117 each in the intervention and control groups. Adopting the cluster sampling method, we recruited 253 children: 127 in the intervention arm and 126 in the control arm.

### Cluster randomization and intervention allocation

**Fig. 1:**
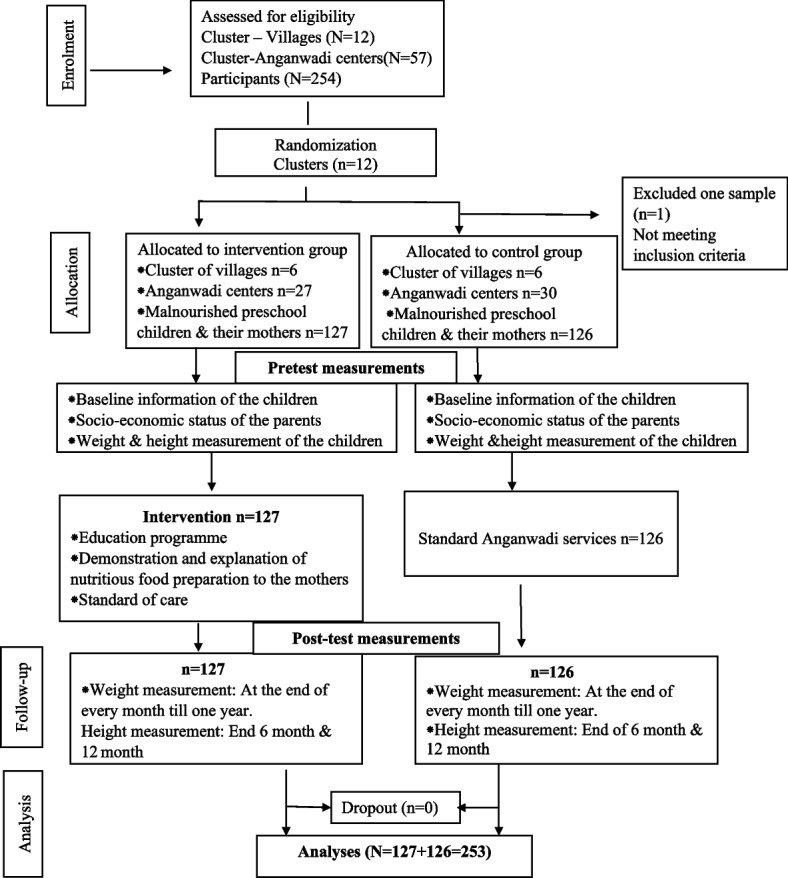
CONSORT (2010) flow diagram of the study

The study followed cluster randomization to allocate the subjects for intervention and control groups. There are 106 villages in the district. Considering the average number of children registered in the Anganwadi center and sample size calculation, the study required 12 villages to be selected. The sampling frame of all the villages was prepared and by lottery method, 12 clusters (villages) were allocated to the intervention and control groups (six clusters each) randomly. All the Anganwadi centers belonging to these villages and all malnourished preschool children and their mothers who met inclusion criteria were enrolled in the study. Thus 127 preschool children and their mothers from 27 Anganwadi centers of six villages were chosen for the intervention group, and 126 preschool children and their mothers from 30 Anganwadi centers of six villages were enrolled in the control arm. The schematic presentation of the cRCT followed the guidelines of CONSORT 2010 [12] (Fig. 1).

### Eligibility criteria for the selection of the sample

The preschool children were considered to be eligible if they were between the age group of three and 5 years, with moderately or severely underweight. Preschool children with a known history of systemic illness such as cardiac problems, renal problems, etc. were excluded from the study.

### Primary outcome

The primary outcome of this study was to find the effect of a home-based nutritional intervention program on improving the nutritional status of malnourished children.

### Ethical approval

All methods were carried out in accordance with relevant guidelines and regulations. Permission was obtained from the concerned authorities. The study was approved by Kasturba Hospital Manipal, Institutional Ethics Committee, and registered in the Clinical Trial Registry of India (CTRI/31/03/2017/008273). A detailed subject information sheet was given in the local language (Kannada) to all the mothers and informed consent was obtained from each mother after explaining the purpose and methodology of the study and ensuring the confidentiality of the data obtained.

#### Home-based nutritional intervention program

Intervention was delivered by the investigator. It includes health education and a demonstration of protein-energy-rich recipes. It also contained the development of a module on the home-based management of malnutrition for mothers.

#### Health education

The health teaching included health education and illustration and presentation of several nutritious recipes to the mothers. The contents of the program mainly targeted malnutrition: its meaning, various risk factors for malnutrition, signs, and symptoms, how to identify malnutrition, and grading with the help of a growth chart. It also comprised of detailed explanation of the methods and approaches to prevent malnutrition, management of diarrhea, cold, cough, and fever, and deterrence and avoidance of infection at home setup. The education session also consisted of teaching on balanced diet and dietary requirements of healthy and malnourished preschool children with a sample diet plan for moderately and severely malnourished preschool children. It also included the method of preparation of 15 types of nutritious recipes which are rich in protein and iron, explanation and demonstration of some recipes using ingredients required for the recipes. The recipes explained and demonstrated are locally available, affordable, and acceptable by the local community.

#### Module for mothers on home-based management of malnutrition

The researcher developed a module on the management of malnutrition at home, especially for mothers with the intent to advance the knowledge of the mothers by reading the module and aiding to prepare nutritious protein-energy food for their malnourished child. The module included information on causes, screening, weight plotting on a growth chart, and management of malnutrition. It comprised information regarding the control of infection, the practice of good hygiene, management of diarrhea, recurrent cold and cough, and worm infestation, with a distinct, in-depth and simple explanation: by dealing in detail with the dietary requirements for preschool children, advice on children’s meals, and also a sample diet plan for a day for moderate and severely malnourished children. It also consisted of the method of preparation, the ingredients required for the recipe, and nutritive values of 15 types of energy and protein-rich recipes which are locally available, affordable (cost-effective), culturally appropriate, and practically feasible to prepare at home [13–18].

### Data collection procedure

A cluster of six villages was randomly allocated to the intervention group and another six villages to the control group. All the Anganwadi centers of the selected villages were visited and the weight and height of children who met the inclusion criteria were measured and collected.

We assessed the nutritional status of the children using specific indicators including weight-for-age and height-for-age.

The monitoring was done using a standard calibrated digital weighing scale, kept on a firm horizontal surface to the nearest 500 g with zero error, and height was measured using a wall-mounted measuring tape. The nutritional status of the children was gauged and reviewed, and it was graded according to the new WHO child growth standards (2006). The indicators used for this are underweight (low weight-for-age) and stunting (low height-for-age) based on the relationship with weight, height, and age. The status of the child with regard to being underweight and stunting is determined by the number of standard deviations in measurements below the mean of the NCHS/WHO reference population [19]. The nutritional grade of the children was ascertained, and the moderately malnourished (weight-for-age < −2SD to -3SD z score), severely malnourished (weight-for-age < −3SD) and stunting (−2SD z score) preschool children were identified. The mothers of the malnourished children were met and explained about malnutrition, the purpose of the study, the data collection procedure, intervention, and follow-ups. The Anganwadi teachers coordinated the discussion between the mothers and the investigators. Accordingly, the date and time for intervention at the Anganwadi center were planned. The nature of the study was elaborated in detail, a subject information sheet was given in the local language, and informed consent was obtained. The houses of the preschool children were visited to meet the mothers of those children who could not attend the discussion at the Anganwadi center. Assessed the nutritional status of the children. For the mothers of those malnourished children who were absent during the previous visit, repeat visits were made to the Anganwadi centers. This was performed to avoid the loss of subjects.

At the Anganwadi center, arrangements were made for group meetings for the delivery of the intervention. Socio-demographic [20] information was collected from the mothers and a health teaching session on home-based management of malnutrition was delivered. The mothers were provided education on malnutrition, its causes, signs, and symptoms, how to identify malnutrition, and plotting and grading by using a growth chart with the help of power points, posters, and flipcharts. Detailed description and elucidation were presented, and discussion was done on ways to prevent and manage malnutrition, management of diarrhea, cold, cough, and fever, and also prevention of infection at home set-up. A balanced diet and the dietary requirement of a malnourished child with a sample diet menu plan for moderate and severe malnourished preschool children were also explained. During the session, we highlighted on different recipes containing high protein and energy, the ingredients required, and their nutritive values. The preparation of various nutritious recipes rich in protein, energy, calcium, and iron was also discussed. One of the nutritious recipes i.e., mixed vegetable and gram (paushtic kichdi) was demonstrated. It took three hours to complete the instruction (education) and demonstration. The mothers were provided with a module on home-based management of malnutrition, which, they were advised to read well and follow. They were asked to prepare anyone nutritious food daily (or at least four times per week) and maintain a diary to record this.

For the intervention group, the intervention was provided in addition to the regular services available at the Anganwadi center. For the control group, regular services by the Anganwadi center were provided.

### Follow-up and outcome measurement

During the intervention phase, a health teaching reinforcement was made by the researcher to the mothers during monthly mothers’ meetings at the Anganwadi centers. The queries of the mothers were answered and suggestions were provided when the children had any problems. The diary maintained by the mothers on nutritious diet preparation and dietary practices was checked during the follow-up. The follow-up sessions also enquired about the health and sickness of the children; provided them with appropriate suggestions.

An Outcome assessor, a trained qualified nurse who was blinded about the study group, performed the assessment of the growth parameters of the children. The weight was tested every month for a year (12 months), and height was tested at 6 months and 12 months of the intervention. The same procedure was followed for the control group, meeting mothers and checking weight and height.

### Quality assurance

An independent person by blind allocation carried out the randomization of villages to the intervention and the control group. This is done to avert the bias in allocation concealment. All Anganwadi centres belonging to allocated villages were included to avoid sample contamination, and all malnourished preschool children who met the inclusion criteria from those Anganwadi centers were included in the study. The researcher approached the Anganwadi center as per the concealment allocation of the villages to the intervention group and the control group. A single researcher carried out the pretest measurements and intervention. An outcome assessor, who is a trained person and was blinded about the study group, performed the follow-up and post-test measurements for both the intervention and control groups. The anthropometric measurement was carried out as per WHO recommendations; which included training in measurement techniques, periodic calibration of the equipment, and supervisors (team members) reviewed activities periodically.

### Data analysis

Data from all points of measurement were pooled and analyzed using SPSS version 16. A Chi-square test was carried out to test the homogeneity of baseline outcome variables among the intervention and control groups. Repeated measures ANOVA, were used to test the effectiveness of the intervention.

In repeated measures, ANOVA to check for the assumption of sphericity violation, and Mauchly’s test was used. In case of violation of sphericity, Greenhouse Geisser correction was used (*p* < .05). In order to explore the difference in outcome variables at different time points of measurements, a post-hoc test using Bonferroni correction was computed and pairwise comparison of the intervention and control group was done.

## Results

### Baseline characteristics of enrolled children and their families

Baseline data shows that most of the children in both the groups (intervention 49.6% & control group 62.7%) belonged to the age group of 3.0 to 4.0 years and were females (intervention 53.5% & control group 61.1%). The majority of them belonged to poor socio-economic status (intervention 74.8% & control group 74.6%). A comparison of the sample characteristics of the intervention and control group showed the groups were similar in terms of age, gender, and type of family distribution (Table 1).

**Table 1:** Baseline characteristics of enrolled children and their families

Variables	Intervention group *n* = 127 f (%)	Control group *n* = 126 f(%)	*p* value
**Age of the child (in years)**			
3.0–4.0	63 (49.6)	79 (62.7%)	0.107
4.1–5.0	49 (38.6)	35 (27.8%)	
5.1–6.0	15 (11.8)	12 (9.5%)	
**Gender of the child**			
Male	59 (46.5%)	49 (38.9%)	0.138
Female	68 (53.5%)	77 (61.1%)	
**Type of family**			
Nuclear	66 (52%)	47 (37.3%)	0.013
Joint	61 (48%)	79 (62.7%)	
**Socio-economic status of the family**			
High	0 (0%)	1 (0.8%)	0.002
Middle	20 (15.7%)	30 (23.8%)	
Poor	95 (74.8%)	94 (74.6%)	
Below poverty line	12 (9.4%)	1 (0.8%)	

### Effect of intervention on the weight of malnourished preschool children

**Fig. 2:**
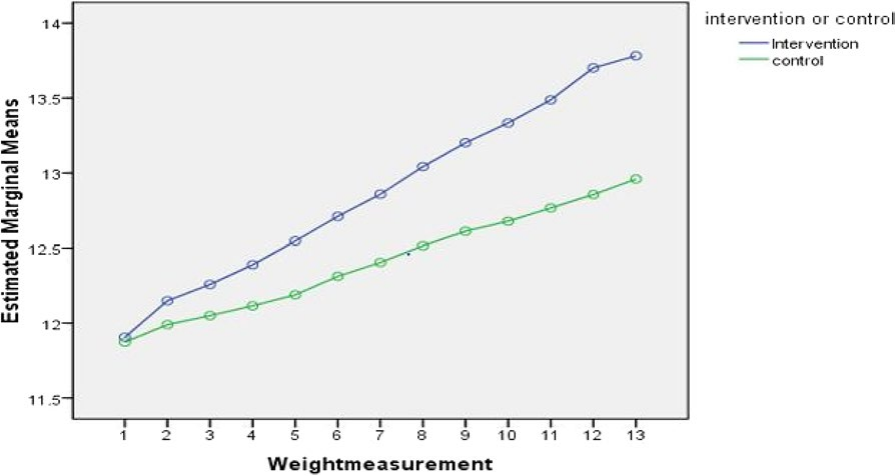
Profile plot showing mean weight of the preschool children

The baseline mean weight in the intervention group changed from 11.9 ± 0.98(kg) to 13.78 ± 0.89(kg) at the end of 12 months whereas in control groups from 11.8 ± 1.03 (kg) to 12.96 ± 0.88(kg). The change in the weight of the children in the intervention group was steady across different time points, while in the control groups the changes were minimal. We could also observe at the end of 1 year (12 months) the mean weight of the intervention group was higher than that of the control group (Table 2 & Fig. 2).

**Table 2:** Pre-test and post-test mean and standard deviation of children’s weight scores in the intervention and control group

Weight of the children (Kilo grams)	Intervention group *n* = 127		Control group *n* = 126	
	Mean	SD	Mean	SD
Pre-test (Baseline)	11.9	0.98	11.8	1.03
Post-test 1	12.1	0.98	11.9	1.0
Post-test 2	12.2	0.98	12.0	1.01
Post-test 3	12.3	0.98	12.1	1.0
Post-test 4	12.5	0.97	12.1	1.0
Post-test 5	12.7	0.96	12.3	0.99
Post-test 6	12.9	0.95	12.4	0.97
Post-test 7	13.0	0.91	12.5	0.95
Post-test 8	13.2	0.90	12.6	0.95
Post-test 9	13.3	0.91	12.6	0.93
Post-test 10	13.49	0.89	12.77	0.91
Post test 11	13.70	1.28	12.86	0.89
Post test 12	13.78	0.89	12.96	0.88

Further the analysis of repeated measures ANOVA between the intervention and control groups showed a statistical significance F _(1, 251)_ = 15.42, *p* < 0 .001, partial eta η_p_^2^ = 0.058, using Greenhouse-Geisser correction. Within the intervention group analysis of repeated measures ANOVA proved a greater statistical significance, F _(12, 125)_ = 974.96, *p* < 0.001, effect size (partial eta) η_p_^2^ = 0.795. Further, repeated measures ANOVA group x time interaction was computed to find the weight gain in the intervention group than the control group. It showed statistical significance F _(12, 251)_ = 69.96, *p* < 0.001 (Table 3).

**Table 3:** Repeated measures ANOVA on weight scores between and within the groups

Weight (kilograms)	Mean square	F-value	df	p	η_p_^2^
Between group (*N* = 253)	177.73	15.42	1, 251	<.001	.058
Within group (*n* = 127)	249.12	974.96	12, 125	<.001	0.795
Group x Time interaction (*N* = 253)	17.87	69.96	12, 251	<.001	0.218

To find out the significant difference in the means across different time points, a post-hoc test using the Bonferroni correction was done. There was mean difference of weight gain across the time points in the intervention group. The increase in weight gain identified from baseline to post-test 1 is 0.180 and from baseline to post-test 12 is 1.480 which is statistically significant at *p* < 0.001. This explains that the mean difference in weight among children in the intervention group was increasing consistently and is statistically significant.

### Effect of intervention on the height of malnourished preschool children

**Fig. 3:**
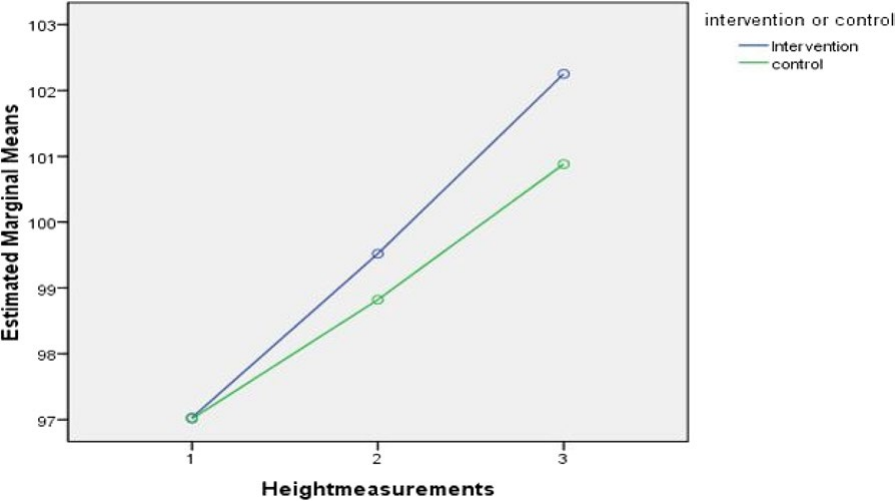
Profile plot showing mean height of the preschool children

The mean scores of the height of the children in the intervention group changed from 97.03 ± 5.42(cm) to 102.2 ± 5.03(cm) whereas in the control group from 97.01 ± 5.79(cm) to 100.88 ± 561(cm). The changes observed from the pretest to the post-test in the intervention group were higher compared to the control group (Table 4 & Fig. 3).

**Table 4:** Mean and standard deviation of pre and post tests height of intervention and control Group

Height of the child (in centimeters)	Intervention group *n* = 127		Control group *n* = 126	
	Mean	SD	Mean	SD
Pre-test (baseline)	97.03	5.42	97.01	5.79
Post test 1	99.52	5.19	98.82	5.67
Post test 2	102.2	5.03	100.88	5.61

Analysis within the intervention group proved a statistical significance F _(2, 1258_) = 1.540, *p* < .001, η_p_^2^ = 0.860. The variance between the intervention group and control group was not found to be statistically significant F _(1,251)_, 1.04, *p* = 0 .308, η_p_^2^ = 0 .004 (Table 5).

**Table 5:** Repeated measures ANOVA on height measurement between and within the groups. *N* = 253

Height (centimeters)	Mean square	F-value	df	P	η_p_^2^
Between group (*N* = 253)	91.646	1.04	1, 251	.308	0.004
Within group (*n* = 127)	1752.84	1.54	2, 125	.001	0.860
Group x Time interaction (*N* = 253)	38.90	34.33	2, 251	.001	

Post-hoc test using Bonferroni correction was done to find out the significant difference in the means at different time points and it revealed, the increase in the mean difference of height from the baseline to posttest 1 in the intervention group was 2.15 cm and baseline and post-test 2 was 4.54 cm which is statistically significant at *p* < .001. Height gain in the intervention group at different point times was statistically significant (*p* < .0001).

### Comparison of the pretest and posttest nutritional status based on WAZ score in intervention and control group

Data in Table 6, presents the effectiveness of the intervention on weight gain in terms of weight-for-age Z scores. In the intervention group, by the end of 1 year, the percentage of preschool children with moderate malnutrition (> − 2SD—3SD) decreased from 41.5 to 24%, severe malnutrition from 8.69 to 3.16% and 20.5% of malnourished children achieved normal nutritional status by 1 year of the intervention. In the control group, the malnourished children had minimal changes in nutritional status (Table 6).

**Table 6:** Effectiveness of nutrional intervention in terms of weight-for-age Z scores

Nutritional status	Intervention group		Control group	
	Baseline f (%)	Post-test 12 f (%)	Baseline f (%)	Post-test 12 f (%)
Moderate malnutrition				
> − 2SD—3SD	105 (41.5%)	63 (24%)	110 (43.47%)	105 (41.5%)
Severe malnutrition				
> − 3SD	22 (8.69%)	8 (3.16%)	16 (6.3%)	13 (5.13%)
< −2SD	0	56 (20.5%)	0	8 (3.16%)

## Discussion

Using a cluster randomized trial this study demonstrated that, the impact of a home-based nutritious diet for malnutrition management proved effective to improve the nutritional status of malnourished preschool children. The intervention was unique as the trial focused on empowering mothers of malnourished underweight preschool children which provided an opportunity to combine behavior change in the form of knowledge improvement that promotes health, care practices during illness and adequate nutrition access to products that support the food-insecure households in providing a nutritionally adequate diet to their young children. The change in weight of the children in the intervention group was a steady improvement across different time points. There were significant weight gain and an increase in height among the children in the intervention group. A higher number of children attained normal nutritional status in the intervention group compared to the control group. There was a substantial improvement in the nutritional status of the children in the intervention group as against the control group.

During the early 2000s, the World Health Organization (WHO) adopted the community-based management of acute malnutrition (CMAM) model and recommended ready-to-use therapeutic-food (RUTF) intervention to enhance the nutrition intake of children suffering from severe acute malnutrition (SAM) [21]. Since 2007, the WHO recommends RUTF for home-based management of uncomplicated SAM to cope with their routine nutritional requirements and support catch-up growth. Also, the WHO recommended that such RUTF should be produced locally by each country, keeping in view the International Standard [22].

Although WHO recommends the CMAM model its efficacy remains suboptimal due to various reasons in many countries. The acceptance of this recommendation has been limited in countries like India. The RUTF strategy for the management of malnutrition does not seem to be logistically feasible due to the complex interplay between poverty, social exclusion, and in-patient treatment with unacceptable costs to the family [23].

Developing countries like India have a large number of malnourished children with a large proportion of them living in rural areas. Their parents lack proper resources, and high costs, which is unacceptable to the family. Delayed institutional management may lead to increased morbidity and mortality. Considering the current scenario, good dietary practice and convincing communication is the right way to correct the problems [10].

The result of the study was broadly consistent with previous studies on the effect of interventions that promoted dietary practices and nutritional status of the children through behavioral change communication. A randomized controlled trial in Uttar Pradesh, India (2016) compared the WHO-recommended therapeutic food versus home-based food in the management of severe malnutrition. The mean rate of weight gain, gain in height, and increase in mid-upper arm circumference showed significantly higher (*p* < .05) in the group that received home-based therapeutic food. The mean duration to achieve target weight (normal nutritional status) took lesser time in malnourished children who consumed a home-based therapeutic diet than in RUTF. The home-based food is more acceptable in terms of improved palatability, additional affordability, increased frequency of feeding, and less difficulty in preparing home-based therapeutic food [10]. Home-prepared Khichri (rice and green gram gruel) was well accepted against RTUF. Khichuri is a palatable meal and is culturally acceptable and widely consumed by families and malnourished children [24].

Many compared hospital management and home strategies for malnutrition children involving interventions in different combinations. Studies have found that a home-based diet is better than hospital management for malnutrition. Energy-dense therapeutic diets with low bulk are vital. It should be made economical, available, and acceptable. These home-based therapeutic diets could be prepared at home from the household pot. In a family, frequent feeding should be viable (6 to 8 times per 24 hours) and can maintain hygiene as well. Commercially accessible international RUTF may not be suitable (acceptable, cost-effective, and sustainable) for Indian settings with uncomplicated SAM [25]. There was an acceleration in weight gain among malnourished children who received a home-based diet and weight gain was sustained as the time of home-based diet therapy extends. Home-based diet therapy is more effective, acceptable, feasible, and practical [26]. It can be concluded that home-based diet management could be a feasible, acceptable, and cost-effective option for malnourished children.

In our study, 20.5% of the study population achieved normal nutritional status from malnourished status in the intervention group, whereas in the control arm only 3.16% and also the proportion of severely underweight reduction was greater than the control arm. Larger number of malnourished children reached normal nutrition status and shifting from severe to mild malnutrition status was higher in the intervention group after 6 months of intervention [26]. It has proved appropriate information makes the mother develop comprehensive care practices to remain their children healthy.

It is essential to comprehend that mothers/caregivers of malnourished children mostly come from low socioeconomic status and have difficulty leaving home for a long period during the treatment of malnutrition. If under-five children are identified in an early stage, home-based food therapy will be simpler, and cheaper with adequate quantity and quality of food resulting in a high success rate in reducing malnutrition, and low costs for the management of malnutrition. It also benefits the mothers spending their time with children and family. It also kindles or promotes the involvement of nutritious food preparation and interest in taking care of the child during sickness.

It was strongly felt that, along with existing health and nutrition services such as ICDS, RCH II, IMNCI, and National Health Mission to the child with malnutrition, there is a need to educate and empower mothers. It should provide nutritional counseling to mothers of malnourished children. The health care provider should strengthen this counseling and endorse home-based food which is small, frequent, and energy-dense feeds. The content of the communication needs to be simple, appealing, technically correct, culturally acceptable, and practical. These messages need to be backed up by appropriate services.

### Strength of the study

Cluster randomized controlled trial conducted with intervention and multiple posttests and a one-year follow-up. Repeated follow-up and family visits made the sample mortality zero. The mothers in this study reported and maintained a diary on nutritious food prepared for their children and nutritious feeding photos were taken which proved compliance with the intervention. The intervention focused on the preparation of nutritious recipes at home and maintaining hygienic practices while preparing recipes, and feeding the child daily.

### Limitations of the study

The study included only preschool children and their mothers registered in Anganwadi centres of selected villages of Udupi taluk. It may be helpful to identify a larger population the result of this study may be generalized to under-five children. Compliance of intervention is depending on the mother’s report and diary maintained by the mother and directly not observed. The influence of socioeconomic status on of the family intervention and dietary intake of the children were not assessed before and after the intervention.

## Conclusion

The nutritional status of preschoolers was found to be significantly improved by home-based intervention. Since home-based diet therapy includes the caregiver’s involvement in preparing a nutritious diet, it leads to a healthy diet practice in the family thereby reducing malnutrition in children. Policymakers must be informed about the current nutritional condition of under-five children and be reminded that a home-based diet is a more affordable therapeutic option that lowers childhood morbidity and mortality. The country needs multidimensional strategies to lessen the nutritional problems, given the geographic, cultural, and financial situation that exist across the country; protein and energy-dense therapeutic home-based diet management are probable to be one of them. The present study findings can be utilized at a larger scale across the country and also among the resource limited settings.

## Abbreviations

ANOVA: Analysis of Variance
CONSORT: Consolidated Standards for Reporting Trials
CMAM: Community-based Management of Acute Malnutrition
CTRI: Clinical Trial Registry of India
ICDS: Integrated Child Development Services
IMNCI: Integrated Management pf Neonatal Child Illness
NCHS: National Center for Health and Statistics
RCH: Reproductive and Child Health
RUTF: Ready-to-Use Therapeutic Food
SAM: Severe Acute Malnutrition
SD: Standard Deviation
SDG: Sustainable Development Goals
SPSS: Statistical Package for Social Sciences
UN: United Nations
